# How much will it cost to eradicate lymphatic filariasis? An analysis of the financial and economic costs of intensified efforts against lymphatic filariasis

**DOI:** 10.1371/journal.pntd.0005934

**Published:** 2017-09-26

**Authors:** Randee J. Kastner, Elisa Sicuri, Christopher M. Stone, Gabriel Matwale, Ambrose Onapa, Fabrizio Tediosi

**Affiliations:** 1 Swiss Tropical and Public Health Institute, Basel, Switzerland; 2 University of Basel, Basel, Switzerland; 3 ISGlobal, Barcelona Centre for International Health Research (CRESIB), Hospital Clinic - Universitat de Barcelona, Barcelona, Spain; 4 Vector Control Division, Ministry of Health, Kampala, Uganda; 5 RTI International, Kampala, Uganda; New York Blood Center, UNITED STATES

## Abstract

**Introduction:**

Lymphatic filariasis (LF), a neglected tropical disease (NTD) preventable through mass drug administration (MDA), is one of six diseases deemed possibly eradicable. Previously we developed one LF elimination scenario, which assumes MDA scale-up to continue in all countries that have previously undertaken MDA. In contrast, our three previously developed eradication scenarios assume all LF endemic countries will undertake MDA at an average (eradication I), fast (eradication II), or instantaneous (eradication III) rate of scale-up. In this analysis we use a micro-costing model to project the financial and economic costs of each of these scenarios in order to provide evidence to decision makers about the investment required to eliminate and eradicate LF.

**Methodology/Key findings:**

Costing was undertaken from a health system perspective, with all results expressed in 2012 US dollars (USD). A discount rate of 3% was applied to calculate the net present value of future costs. Prospective NTD budgets from LF endemic countries were reviewed to preliminarily determine activities and resources necessary to undertake a program to eliminate LF at a country level. In consultation with LF program experts, activities and resources were further reviewed and a refined list of activities and necessary resources, along with their associated quantities and costs, were determined and grouped into the following activities: advocacy and communication, capacity strengthening, coordination and strengthening partnerships, data management, ongoing surveillance, monitoring and supervision, drug delivery, and administration. The costs of mapping and undertaking transmission assessment surveys and the value of donated drugs and volunteer time were also accounted for. Using previously developed scenarios and deterministic estimates of MDA duration, the financial and economic costs of interrupting LF transmission under varying rates of MDA scale-up were then modelled using a micro-costing approach. The elimination scenario, which includes countries that previously undertook MDA, is estimated to cost 929 million USD (95% Credible Interval: 884m-972m). Proceeding to eradication is anticipated to require a higher financial investment, estimated at 1.24 billion USD (1.17bn-1.30bn) in the eradication III scenario (immediate scale-up), with eradication II (intensified scale-up) projected at 1.27 billion USD (1.21bn-1.33bn), and eradication I (slow scale-up) estimated at 1.29 billion USD (1.23bn-1.34bn). The economic costs of the eradication III scenario are estimated at approximately 7.57 billion USD (7.12bn-7.94bn), while the elimination scenario is projected to have an economic cost of 5.21 billion USD (4.91bn-5.45bn). Countries in the AFRO region will require the greatest investment to reach elimination or eradication, but also stand to gain the most in cost savings. Across all scenarios, capacity strengthening and advocacy and communication represent the greatest financial costs, whereas mapping, post-MDA surveillance, and administration comprise the least.

**Conclusions/Significance:**

Though challenging to implement, our results indicate that financial and economic savings are greatest under the eradication III scenario. Thus, if eradication for LF is the objective, accelerated scale-up is projected to be the best investment.

## Author summary

Lymphatic filariasis (LF) is a neglected tropical disease (NTD) that is targeted for elimination and is thought to be potentially eradicable through once yearly mass drug administration (MDA) using drugs that are currently donated to LF endemic countries by the pharmaceutical companies that manufacture them. MDA has proven to be a cost-effective and efficient method of disease control, both for LF and other NTDs. Previously, we developed scale-up scenarios of varying magnitude to reach elimination (elimination of LF in all countries that have previously undertaken MDA) and eradication (local elimination of LF in all LF endemic countries) and estimated the number of associated treatments that would be necessary in each country under each scenario. Here we project the costs—both financial and economic—of each of these scenarios. We use data from a myriad of sources to estimate the cost of various activities, and found that training and advocacy and communication activities comprise the bulk of the costs of the program. Among all scenarios, elimination requires the least total investment. However, in terms of LF eradication, faster rates of MDA scale-up are associated with decreased overall costs.

## Introduction

Neglected tropical diseases (NTDs) are a heterogeneous group of helminthic, bacterial, viral, fungal and protozoan infections that cause chronic and debilitating disability [1]. However, research and development to combat NTDs have notoriously been underfunded [2]. NTDs persist in areas where access to clean water, hygienic conditions, and health care are limited. As such, they are most prevalent in low-income countries [1]. Indeed, more than 70% of countries with endemic NTDs are classified as low-income or lower middle-income economies [3]. Infection with an NTD may affect cognitive and physical development and can result in permanent physical disability. Therefore, NTDs do not just coexist in poverty, they further propagate the cycle of poverty by hindering economic potential [4, 5].

Lymphatic filariasis (LF), an NTD, can result in irreversible disability, most often manifested as elephantiasis, lymphedema, and hydrocele [6]. With more than a billion people at-risk and 120 million people thought to be infected across 73 countries [7], LF is estimated to account for 2.74 million disability-adjusted life years (DALYs) (1.73m-4.00m) [8]. When incorporating the mild and moderate depression associated with LF-related disability, the health burden due to LF may be upwards of 5 million DALYs [9].

However, while LF can result in profound morbidity, it is inefficiently transmitted, with an estimated 15,500 infective mosquito bites thought necessary to generate one transmittable infection [10]. LF is also preventable through once yearly treatment with antifilarials distributed through mass drug administration (MDA) [6]. This, coupled with the fact that LF does not have a significant animal reservoir, led the International Task Force on Disease Elimination to classify LF as a potentially eradicable disease [11, 12]. In response, the World Health Organization (WHO) began the Global Program to Eliminate LF (GPELF), which aims for the global elimination of LF by 2020 [6]. In the fifteen years since the inception of the GPELF more than five billion antifilarial treatments have been distributed in 58 LF-endemic countries [13].

Successfully eradicating a disease has innumerable long-term health benefits, and is also a classic example of a global public good [14–16]. Eradicating an NTD, like LF, has additional societal benefits, including improvements towards equity, fairness, and social justice [17]. However, disease elimination and eradication initiatives require substantial social and political commitments, as well as significant financial and economic investments. Given the increasingly intense competition for global health resources, the decision on where to invest funds needs to be based upon solid evidence [18]. In order to provide evidence to decision makers about the investment required to eliminate and eradicate LF, we used a micro-costing approach to analyze the financial and economic costs of interrupting LF transmission in all endemic countries under varying levels of MDA intensity, as well as the subsequent costs of conducting post-MDA surveillance.

## Methods

### Scenarios

We previously developed scenarios to reach elimination (elimination of LF transmission in countries that have previously undertaken MDA) and eradication (elimination of LF transmission in all endemic countries) of LF, taking into account previous progress made under the GPELF, pre-intervention prevalence levels, and possible delays in program implementation. The elimination scenario maintains the current geographic expansion and rate of MDA scale-up as seen under the GPELF, and thus serves as the comparator scenario. The eradication scenarios were developed to assess the impact of expanding MDA to all endemic countries at an average rate of MDA scale-up (eradication I), intensifying efforts against LF (eradication II), and treating all endemic populations immediately (eradication III). Key components inherent in each scenario are outlined in S1 Table and a full explanation of all the scenario can be found in Kastner et al [19].

### Timeframe and number of treatments required

To determine the duration of MDA required for the different drug regimens, vector species, and pre-intervention prevalence levels, we used EpiFil, a deterministic model of LF transmission [20]. The amount of time and number of treatments required to reach the endpoints in each scenario are detailed in Kastner et al. [19]. Briefly, we considered the number of MDA rounds that each country had previously achieved a programmatic coverage of at least 65% (the minimum coverage necessary to be considered effective) between 1999 and 2012. Using Epifil, we then determined the expected number of rounds of annual MDA treatments at the level calculated to result in elimination in 97.5% of simulations. Assuming the number of MDA rounds required to interrupt transmission as a deterministic value, we next subtracted the number of rounds of MDA required to reach local elimination from the number of previously effective years. Assuming once-annual MDA (aside from areas co-endemic with hyper Loaisis; see: *Assumptions about L*. *loa endemic areas*), we then determined the number of future treatments needed for each country under each scenario, accounting for the number of people at-risk, country-specific growth rates, duration of MDA necessary, historical rates of scale-up, and previous progress towards local elimination. By assuming that the populations at-risk for LF increase exponentially with population growth rates, scenarios with longer durations were also assumed to require more treatments (see S1 Table).

### Approach used for costing

To assess how much governments and donors would need to invest in order to implement the GPELF strategy to reach the elimination and eradication of LF, we adopted a micro-costing, bottom-up approach from the perspective of the health system of each LF endemic country. In contrast to gross-costing, which assesses average level costs from the top down, micro-costing may improve the accuracy of results by capturing resources and costs at the unit level [21].

The costs associated with each scenario have been assumed to begin in the year 2014 and run until the final post-MDA transmission assessment survey (TAS) has been completed in each country under consideration. All results are listed in 2012 US dollars (USD) and future costs were discounted at 3%. One-way sensitivity on discount rates, variability rates, and probabilistic sensitivity analyses for all costing and quantity parameters were also explored. Two-way sensitivity analyses were also employed to explore the level of interdependence between key parameters and activities.

### Data

Line items from USAID’s *NTD Master Plan Costing Tool in the African Region* for Benin, Cameroon, Democratic Republic of Congo, Eritrea, Guinea, Madagascar, Niger, Senegal, and Sierra Leone ranging from January 2011 to April 2012 were reviewed to preliminarily determine essential activities and associated resources necessary for a country to successfully undertake a program to eliminate lymphatic filariasis (PELF). Subsequently, in consultation with key LF implementers from the PELF in Uganda, which has successfully been carrying out the GPELF MDA strategy since 2002 [13], all activities and resources were further reviewed and a refined list of core activities, necessary personnel, components, and resources, along with their associated costs, were ascertained [22].

Retail prices from established vendors were used for tradable goods, including laboratory supplies and capital items. The WHO CHOICE database was used for unit costs which were unable to be determined elsewhere. While the percentage of time needed for LF personnel was informed by the Ugandan program, the country-specific salary estimates were taken from the WHO CHOICE database for the NTD director (assumed to be 0.2 full time equivalent (FTE), LF program manager (1.0 FTE), administrative assistant (0.5 FTE), finance officer (0.3 FTE), data manager (0.3 FTE), and supplies manager (0.5 FTE). As the African Program for Onchocercaisis Control (APOC) uses a strategy similar to that employed by the GPELF [23], line items in our study were validated against similar line items found in APOC approved budgets.

### Activities considered

We took into account the cost of advocacy and communication; capacity strengthening; coordination and strengthening partnerships; mapping; data management; administration; ongoing surveillance; monitoring, evaluation, and supervision (M&E); drug delivery; and post-MDA transmission assessment surveys (TAS). As described below, the costs of increased surveillance in areas with meso *L*. *loa* prevalence, as well as the added cost of biannual MDA in hyper loaisis areas were also accounted for.

Advocacy and communication was assumed to include the development and distribution of educational messages, as well as community meetings and sensitization activities with district and community leaders, sub-county and parish supervisors, and community drug distributors (CDDs). Capacity strengthening comprised trainings on MDA procedures for national trainers, district trainer of trainers, sub-county and parish supervisors, community leaders, CDDs and teachers. Trainings for monitoring sentinel and spot check sites as well as trainings for M&E officers were also considered under capacity strengthening. Conference attendance and international exchanges, cross-border meetings for regional strategies towards controlling NTDs, NTD secretariat meetings, and technical committee meetings were assumed under coordination and strengthening partnerships. Data management included all activities involved with the acquisition and distribution of MDA data, including cleaning, entering, and analyzing. The maintenance of sentinel sites—including equipment for administering microfilaria (mf) surveys and internal quality control tests—as well as the administration of sentinel and control site impact assessment surveys, associated data collection, and survey feedback meetings were grouped under ongoing surveillance. M&E included the supervision of MDA activities, monitoring for severe adverse events (SAEs), and regular feedback meetings at the district and national level. Drug delivery involved drug transport from the central stores to district stores and then onward to parish supervisors. Supplies to CDDs, including t-shirts and stationery, were also accounted for under drug delivery. Administration included overhead costs, the maintenance of office space, salaries to LF staff, and the procurement of necessary equipment.

Mapping and TAS were assumed to include a preliminary visit, immunochromatographic card test (ICT) testing, data collection, and feedback meetings. The cost of mapping was included for any country that, as of 2012, had not yet completed mapping nor started MDA. In order to determine the costs of post-MDA surveillance on a global level, all TAS have been assumed to be school-based. Moreover, TAS have been assumed to occur in each district (see *Determination of resource quantities* for assumptions about district size) after the final estimated round of MDA (as determined through earlier modeling exercises) and twice thereafter at three year intervals. The number of TAS conducted has thus been assumed to vary by the number of districts achieving the specified number of MDA rounds, though the quantities of resources required for each individual TAS was assumed to remain constant.

### Assumptions about *Loa loa* endemic areas

Previous studies indicate that individuals harboring more than 30,000 *L*. *loa* microfilaria per milliliter of blood are put at unacceptably high risk of developing severe adverse events (SAEs) if administered ivermectin or diethylcarbamazine citrate (DEC) [24–26]. As *L*. *loa* prevalence within a community has been shown to have a close correlation with individual *L*. *loa* mf density, provisional GPELF guidelines recommend communities endemic with LF that also have a *L*. *loa* prevalence greater than 40% be treated with bi-annual MDA using albendazole monotherapy coupled with vector control [27].

Mapping studies to determine areas of co-endemicity between LF and *L*. *loa*, however, are not yet complete. While we recognize that not all populations at-risk for *L*. *loa* are also at-risk for LF, for the purposes of this study, we make the assumption that the percentage of mapped areas from RAPLOA studies that were found to have 20–39.9% (meso) or >40% (hyper) *L*. *loa* prevalence corresponds directly to the percentage of the population in these countries also at risk for LF [13, 28]. In assessing the costs for undertaking the LF program in these areas, we further assume the cost of vector control to be covered by other initiatives. In line with the provisional recommendations, we assume that the population living in hyper-endemic areas will receive bi-annual albendazole through MDA. Financially, this is assumed to double the costs of data management and drug delivery in these areas. For populations in meso-endemic regions of *L*. *loa*, once yearly albendazole and ivermectin is still presumed. In areas of both hyper and meso *L*. *loa*, the costs associated with monitoring for SAEs are assumed to increase two-fold.

### Determination of resource quantities

In line with the approach for assessing necessary activities, quantities and duration of use for each required component were established through consultation with key members from the Ugandan PELF team. Aside from program activities with inherently fixed costs—such as the creation and dissemination of health messages, coordination and strengthening of partnerships, and administrative costs—budgeted line items were assumed to vary linearly by the size of the population to be treated (see below: *Timeframe and number of treatments required)*. In the baseline analysis, we have assumed that the number of resources required to carry out the PELF for a certain population in Uganda is relatively similar to the number of resources required to carry out the program for a population of similar size in other LF endemic countries. As MDA in Uganda is implemented at a community level, the amount of resources and duration of activities required to successfully complete the PELF in Uganda are generally organized by district, sub-county, and village units. In order to standardize the at-risk population falling into the different administrative levels (districts, sub-counties, and villages) across all LF endemic countries, the average number of people at-risk for LF in each district, sub-county, and village were determined for Uganda and then assumed for all LF-endemic countries.

### Determination of distances and transport costs

We assumed the costs to transport both people and supplies within each LF endemic country to vary by a function of distance traveled and country-specific petrol prices. Using NTD national control program plans from 22 LF endemic countries, we estimated the driving distances between each country’s capital city and each of their districts. We then averaged these results and related them to the surface area of the respective country. The resulting average for the 22 countries was then used to relate the surface area of the other LF endemic countries for which NTD national control program plans were unavailable to intra-country distances. Distance estimates were then paired against the median price of 1 liter of petrol in each LF endemic country, as reported by Numero in order to estimate transport costs.

### Determination of financial costs

In 2013, the median GDP per capita among LF endemic countries was estimated at 1,245.51 USD, ranging from 226.46 USD in Malawi to 25,140.29 USD in Brunei [29]. Given the highly variable economic conditions across LF endemic countries and lack of expenditure data available for this analysis, we proceeded to estimate unit costs for all local, non-tradable goods and services for each LF endemic country by adjusting detailed expenditure budgets from the LF elimination program in Uganda, a country with a GDP per capita estimated at 571.96 USD [29], by country-specific comparative price levels (i.e., purchasing power parity (PPP) adjusted exchange rates) [30]. Tradable goods were assumed to already be at market value and were thus left unadjusted. Table 1 provides a list of the primary activities considered in calculating the financial costs, as well as the average cost per district in the base case.

**Table 1:** Average costs per district, base case.

Activity	Average costs per district (standard deviation)	Frequency (implementation phase*)
**Advocacy and communication**		
Community meetings	$8,946.96 ($686.11)	Annually
Social mobilization—District leaders	$469.95 ($18.30)	Annually
Social mobilization—Sub-county supervisors	$256.39 ($20.12)	Annually
Social mobilization—Parish supervisors, CDDs, community leaders	$8,821.35 ($753.30)	Annually
Workshop for creating messages	$5.61 ($0.22)	Once every 5 years
Dissemination of health messages	$916.44 ($54.62)	Once every 5 years for printed messages; Annually for verbal messages
**Capacity strengthening**		
Training national trainers, MDA and M&E	$14.31 ($0.54)	Annually
Training of district trainers of trainers, MDA and M&E	$800.35 ($31.22)	Annually
Training of sub-county supervisors, MDA and M&E	$754.87 ($50.60)	Annually
Training of parish supervisors and community leaders, MDA and M&E	$20,991.65 ($2,141.29)	Annually
Training of CDDs	$15,848.40 ($1,655.98)	Annually
Training of teachers	$4,748.92 ($520.45)	Annually
Training for monitoring sentinel and spot check sites	$690.36 ($49.50)	Annually
Training M&E officers	$1,310.23 ($56.96)	Annually
**Coordination and strengthening partnerships**		
Conference attendance and international exchanges	$83.04 ($1.16)	Annually
Attend cross-border meetings for LF and NTDs	$141.62 ($1.69)	Annually
NTD secretariat meeting	$81.81 ($3.35)	Annually
Technical committee of NTDs	$38.28 ($1.71)	Annually
**Data management**		
Cleaning, entering, analyzing data	$119.54 ($6.96)	Annually
Transfer of data from field to head office	$704.69 ($93.39)	Annually
**Ongoing surveillance**		
Maintain sentinel sites	$1,616.73 ($8.80)	Annually
Site survey—data collection	$3,000.23 ($94.92)	Annually
Site survey—feedback meetings	$1,589.09 ($36.39)	Annually
Transmission assessment surveys—preliminary visit	$1,165.08 ($40.04)	Annually
Transmission assessment surveys—data collection	$2,849.99 ($55.82)	Annually
Transmission assessment surveys—feedback meeting	$1,438.37 ($35.32)	Annually
**Monitoring, evaluation, and supervision**		
Supervision of MDA	$18,216.02 ($485.16)	Annually
Feedback meetings at district level	$884.54 ($37.36)	Annually
Feedback meetings at national level	$19.34($0.53)	Annually
**Drug delivery**		
Supplies for CDDs	$3,924.54 ($485.62)	Annually
Drug transport	$3,078.94 ($180.55)	Annually
**Administration**		
Overhead costs	$377.36	Annually
Salaries, LF staff	$950.19 ($51.04)	Annually
Procurement of necessary equipment and software	$61.63 ($2.23)	Annually

*In the base case, the implementation period runs from 2014–2023

### Determination of economic costs

**Fig 1:**
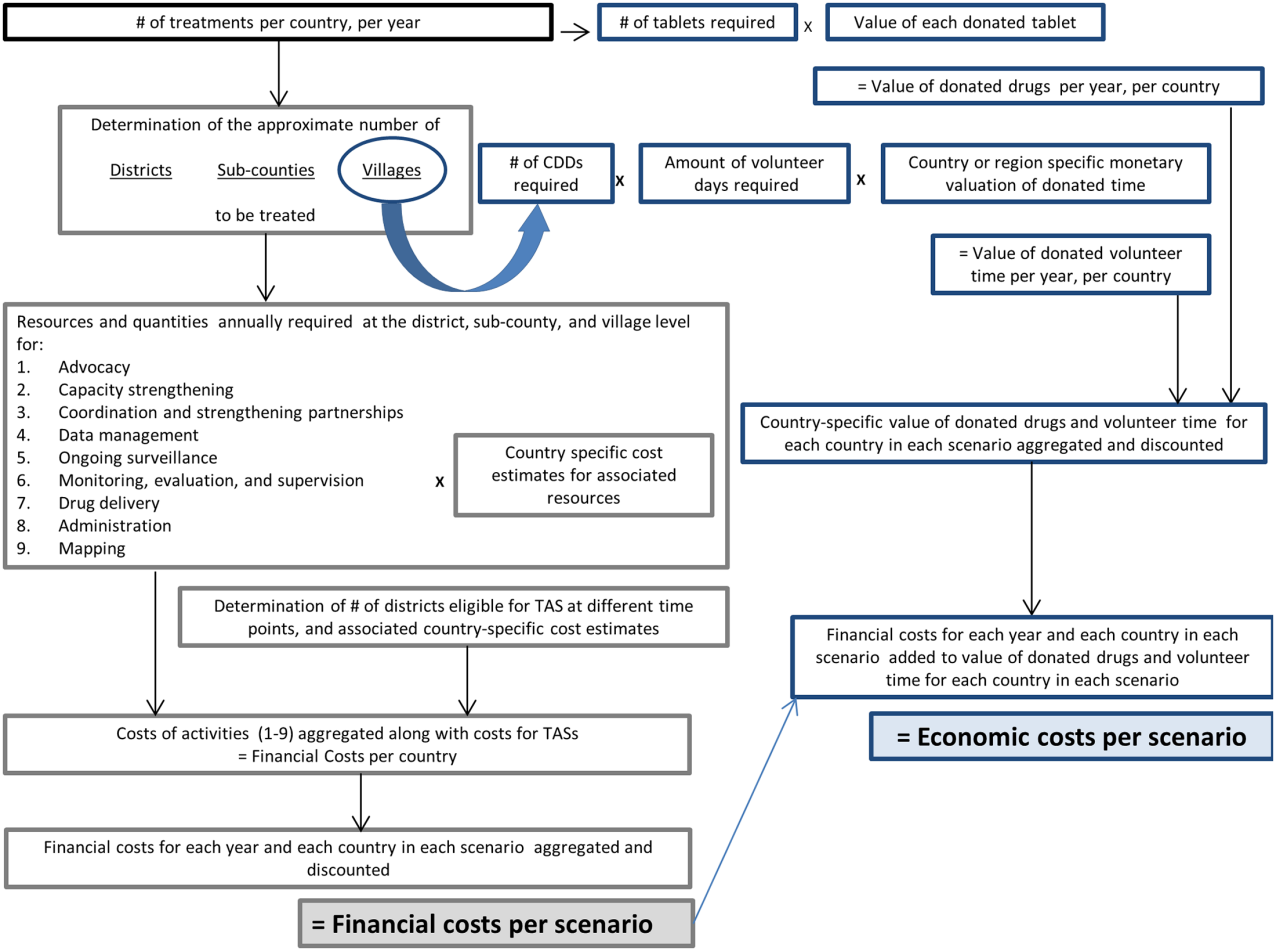
Financial and economic costing algorithm.

Economic costs, which were assumed to encompass financial costs as well as the value of volunteer time and donated pharmaceuticals, were also estimated in order to have a more comprehensive understanding of the projected investment needed to eliminate and eradicate LF [31]. A schematic of the algorithm used for calculating the financial and economic costs is depicted in Fig 1.

#### Value of donated pharmaceuticals

The opportunity costs of the donated drugs used in the GPELF were accounted for by valuing each 400 mg tablet of albendazole at 0.19 USD, 50 mg tablet of DEC at 0.0025 USD, and 3 mg tablet of ivermectin at 0.50 USD, which were the suggested manufacturer prices prior to being donated [32–34]. An additional economic cost of 0.0018 USD was assumed to the value of each tablet for insurance and shipping costs, which also are currently absorbed by the drug manufacturers [34]. While the WHO specifies each treatment to include either 6 mg DEC/kg of body weight or 150 μg ivermectin/kg of body weight plus 400 mg ALB, for the purposes of this global level exercise, we assume all annual MDA treatments to be comprised of one tablet of ALB with either three tablets of ivermectin or seven tablets of DEC.

#### Value of volunteer time

The value of donated time was evaluated by correlating the time CDDs were presumed to volunteer under each scenario with country-specific or, when necessary, region-specific daily per worker agriculture wage estimates taken from the World Bank’s World Development Indicators Online, inflated to 2012 [35]. Two CDDs were assumed to be sufficient to dispense MDA in each village [36]. Drawing from the results of previous time studies, CDDs were assumed to volunteer 5.5 days on mobilization and sensitization, 4.6 days conducting pre-MDA census activities, and 17.8 days on drug distribution [37].

### Uncertainty analysis

To account for the uncertainty in our model parameters, and to examine the affect of this uncertainty on the outcome and conclusions of our study, we conducted a series of uncertainty analyses. For the primary analysis, we conducted a probabilistic sensitivity analysis (PSA) involving all financial unit costs and quantities assuming 10% uncertainty and gamma distributions for all parameters in order to avoid negative values [38]. As the covariance between parameters was unknown, we further assumed all parameters to be independent. For all scenarios, we ran the model for 500 iterations for every year in every country. The model outputs thus provide a distribution of cost results, reported as median estimates and associated 95% credible intervals. Additional details involved in conducting the PSA can be found in the S1 File. Additional sensitivity analyses, including the impact of assuming 30% uncertainty of all input parameters, as well as a series of two-way sensitivity analyses to assess possible correlation between parameters can also be found in the supplementary material (S1 File, S2 and S3 Figs, S3, S4 and S5 Tables).

## Results

**Fig 2:**
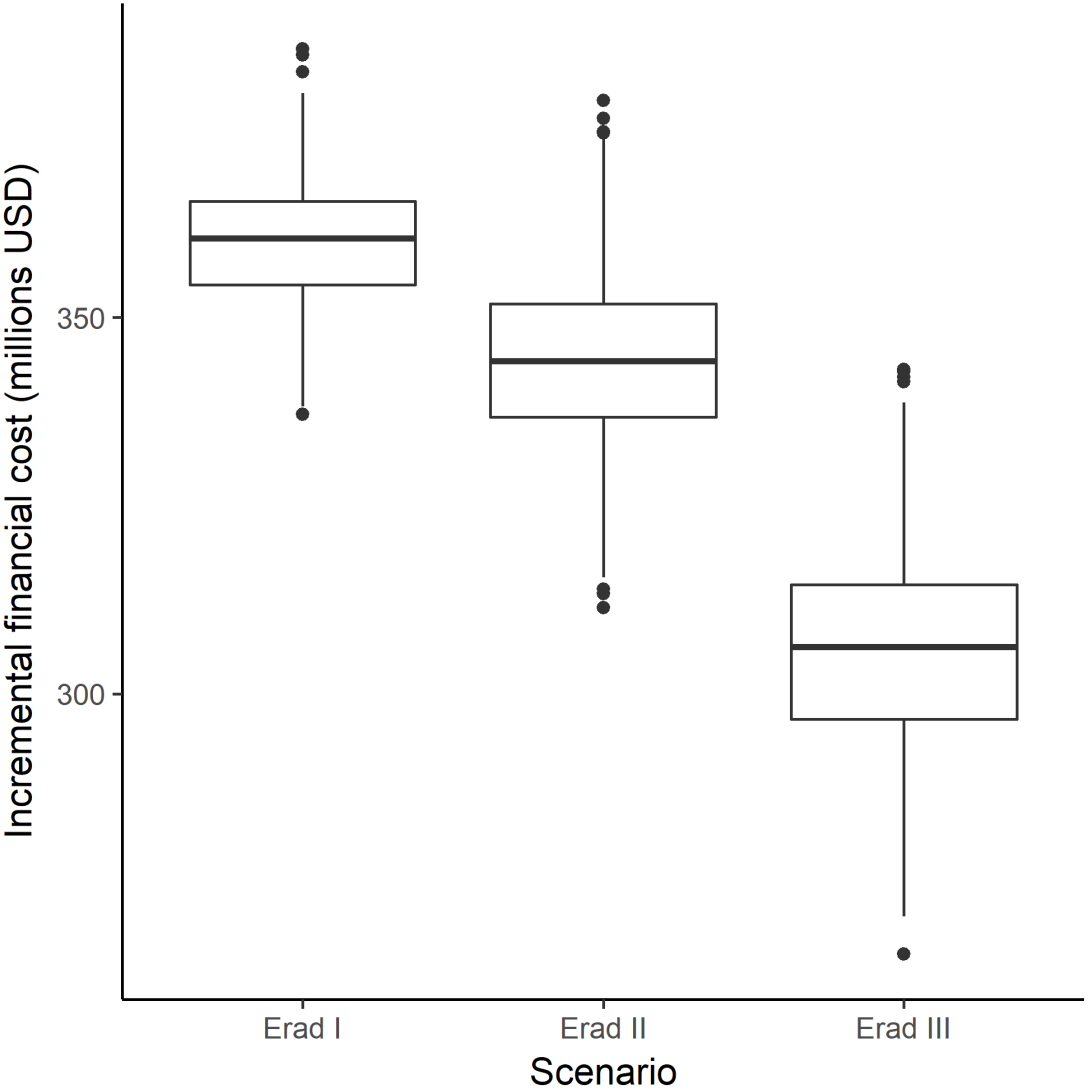
Incremental financial costs (elimination scenario as comparator).

The total financial investment to implement the elimination scenario is projected at 929 million USD (891m-965m). To expand the campaign to all endemic countries at an average rate of scale-up (eradication I scenario) would require 1.29 billion USD (1.24bn-1.34bn), an increase of about 360 million USD (346m-374m) over the elimination scenario (Fig 2). The decrease in scenario duration inherent in the eradication II scenario (intensified scale-up) comes with decreased costs, estimated at 1.27 billion USD (1.22bn-1.32bn), while instantaneously scaling up MDA to all LF endemic countries is projected to require an investment of 1.24 billion USD (1.18bn-1.28bn). The AFRO region accounts for 62–68% of the financial costs, with Southeast Asia requiring between 22–30% of the projected investment (Table 2).

**Table 2:** Total financial costs by region (in millions USD).

	Elimination (comparator)	Eradication I	Eradication II	Eradication III
AFRO	$ 574m ($547m-$600m)	$ 878m ($841m-$915m)	$ 871m ($833m-$907m)	$ 840m ($802m-$877)
SEAR	$ 278m ($266m-$289m)	$ 278m ($266m-$289m)	$ 279m ($267m-$292m)	$ 279m ($266m-$292m)
WPR	$ 56m ($55m-$58m)	$ 67m ($65m-$68m)	$ 58m ($56m-$59m)	$ 54m ($53m-$56m)
AMR	$ 21m ($20m-$22m)	$ 21m ($20m-$22m)	$ 19m ($18m-$20m)	$ 18m ($18m-$19m)
EMR	$0.44m ($0.43m-$0.45m)	$ 46m ($44m-$48m)	$ 47m ($44m-$49m)	$ 43m ($40m-$46m)
Total Financial Cost	**$ 929m** ($891m-$965m)	**$ 1,289m** (1,239m-1,337m)	**$ 1,273m** (1,223m-1,322m)	**$ 1,235m** (1,183m-1,284m)
Total Economic Cost	**$ 5,187m** ($4,907m–$5,450m)	**$ 7,915m** ($7,498m–$8,300m)	**$ 7,975m** ($7,547m–$8,366m)	**$ 7,532m** ($7,117m–$7,937m)

**Fig 3:**
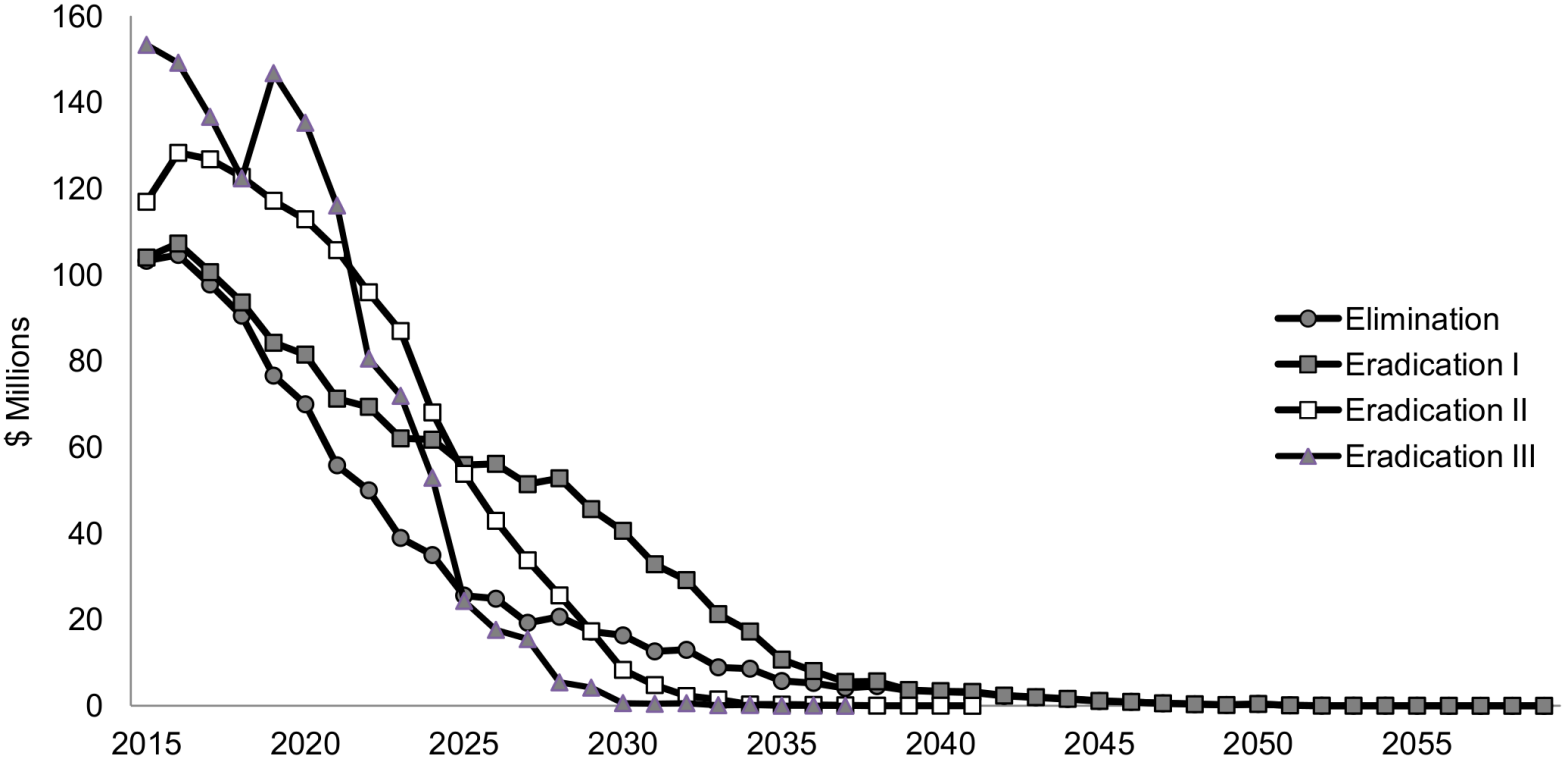
Financial costs by year, discounted at 3%.

Providing MDA to the entire at-risk population immediately, as assumed under the eradication III scenario, requires a significant initial investment, but within 10 years’ time, the annual cost of implementing the scenario becomes less than the alternatives (Fig 3). The sharp increase in financial costs four years from the start of the eradication III scenario corresponds to the start of MDA to all at-risk populations in countries that were previously delayed due to mapping.

**Fig 4:**
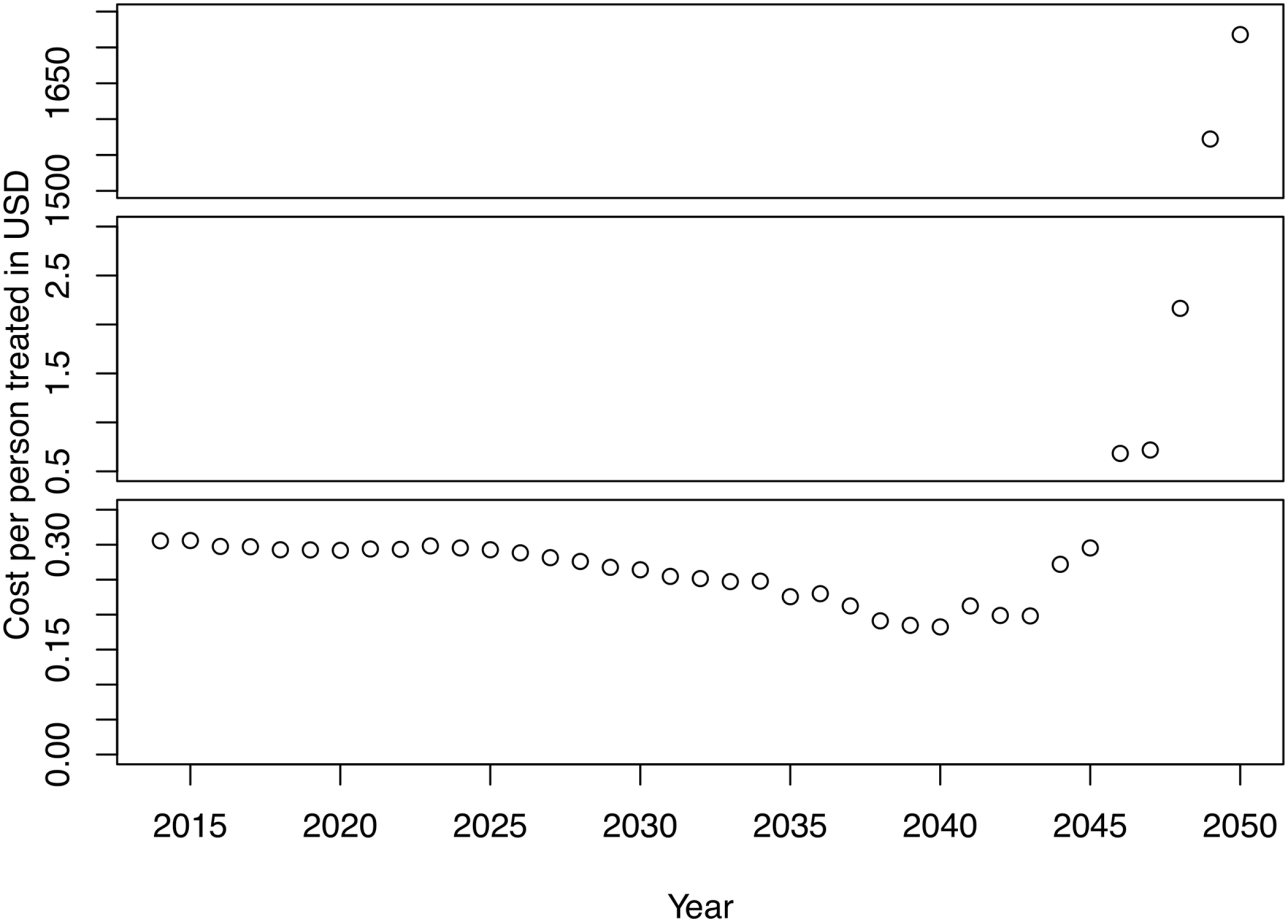
Financial cost per person treated, eradication I.

The average unit financial cost for undertaking each of the scenarios ranges from 0.27 USD in the elimination scenario to 0.31 USD in both the eradication II and III scenarios. However, as the scenarios progress, the unit costs increase substantially. This is due to the fact that the number of people to be treated (the denominator of the estimate) decreases, though the cost associated with some of the core activities—including coordination and strengthening partnerships, administration, and data management—are assumed to remain relatively constant. As an example of this, Fig 4 depicts the unit financial costs seen under eradication I, which, by 2050, extend to more than 1,700 USD per person treated.

Capacity strengthening proves to be the most costly activity, representing between 53–55% of the overall financial costs, while advocacy and communication (22–24%); ongoing surveillance (6%); monitoring, evaluation, and supervision (8%); and drug delivery (9%) account for most of the remaining costs (Table 3).

**Table 3:** Percentage of financial costs by activity.

	Elimination	Erad I	Erad II	Erad III
Advocacy and communication	24%	22%	23%	24%
Capacity strengthening	53%	55%	55%	53%
Coordination and strengthening partnerships	<1%	<1%	<1%	<1%
Data management	<1%	1%	1%	1%
Ongoing surveillance	6%	6%	6%	6%
Monitoring, evaluation, and supervision	8%	8%	8%	8%
Drug delivery	9%	9%	9%	9%
Post MDA Surveillance	<1%	<1%	<1%	<1%
Administration	<1%	<1%	<1%	<1%
Mapping	-	<1%	<1%	<1%

Our assessment of the financial costs of treating a population of 1 million at-risk for LF with and without *L*. *loa* coendemicity in the Democratic Republic of Congo indicates that areas of meso *L*. *loa* are anticipated to only result in an increase in monitoring and evaluation, thereby having little effect on the overall costs. In comparison to a population of comparable size without *L*. *loa*, hyper *L*. *loa* endemicity is associated with an increase of approximately 15% in the overall costs of the program due to the increase in data management, drug delivery, and monitoring and evaluation activities.

**Fig 5:**
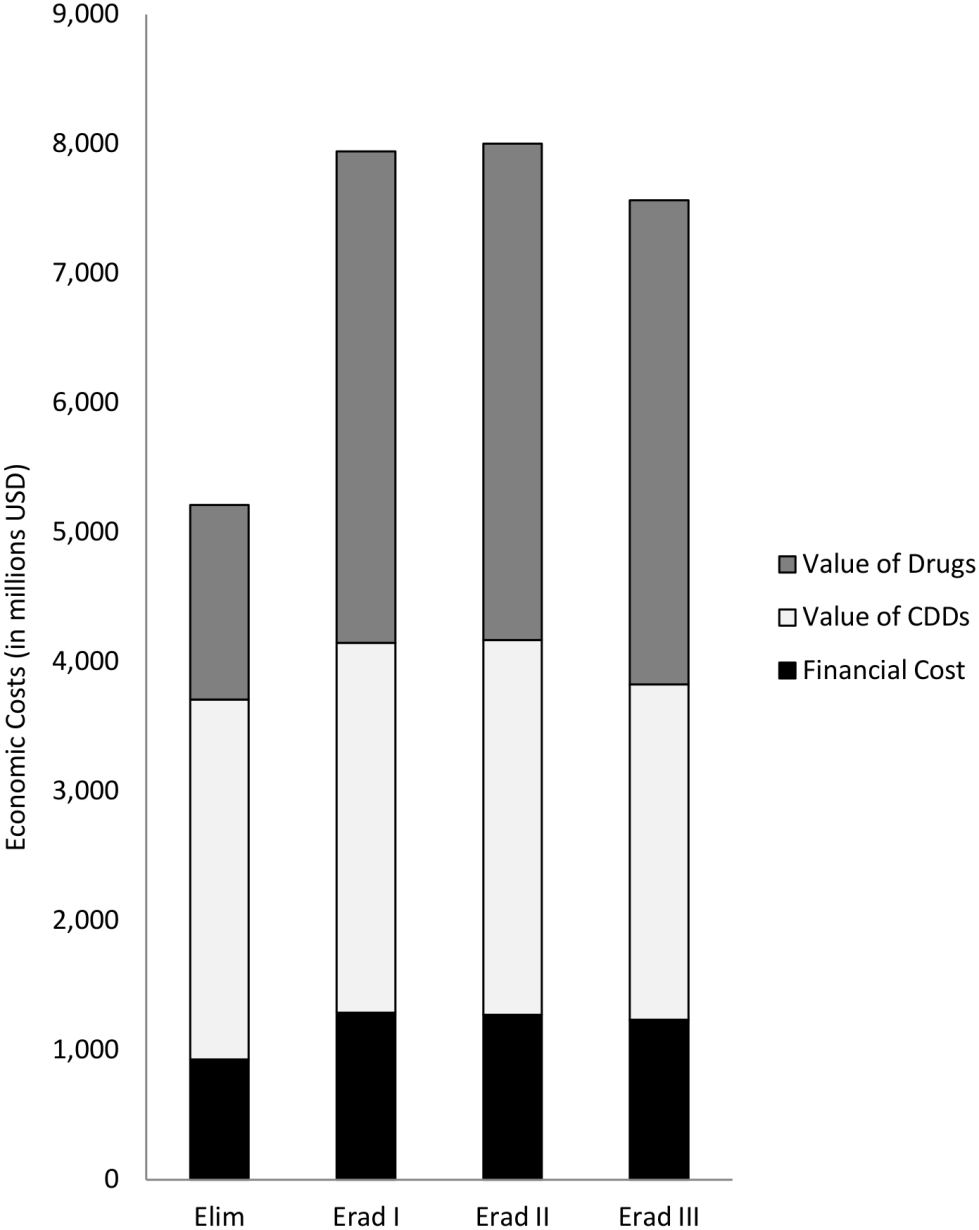
Economic costs by component, discounted at 3%.

When the economic costs are considered, the costs of all scenarios are substantially higher (5.2 billion USD for the elimination scenario). Extending the coverage to all endemic countries is estimated to require around 7.9 billion USD (7.5bn–8.0bn), or 45% more than under the elimination scenario (Table 2). Depending on the scenario, between 48–53% of the economic costs are due to the value of the donated drugs (Fig 5).

## Discussion

This study used a micro-costing approach to provide estimates on the financial and economic investments required to eliminate and eradicate lymphatic filariasis. Across the eradication scenarios, faster rates of MDA scale-up are associated with decreased costs (1.2 billion USD in the eradication III scenario versus 1.3 billion USD in the eradication I scenario). The projected economic costs of eradication range from 7.5 billion to 8.0 billion USD, about half of which is due to the value of the donated drugs. These results serve to further highlight both the importance of higher rates of MDA scale-up as well as the crucial partnership between the GPELF and the drug donation programs. These projections may also be important in order to convince pharmaceutical companies to continue donating the drugs necessary to eliminate LF. While the cost to eliminate LF is less than that to eradicate, it must be recognized that deciding to pursue elimination rather than eradication signifies the continuation of LF-related costs indefinitely, and comes at a health burden to populations that remain untreated.

With a dearth of evidence on the costs of implementing morbidity management programs [39], and given that the aim of our study was to assess the costs of interrupting transmission of the causative agent of LF, we did not include the costs of morbidity management in our estimates. Had morbidity management been included in the scenarios, the overall costs would have certainly increased, though presumably with reduced times to eradication associated with reduced morbidity management costs.

Our cost estimates for reaching LF elimination and eradication inherently assume that the GPELF strategy, if carried out sufficiently, will lead to the interruption of LF transmission in all areas. In our analysis we have also assumed that accelerating the rate of MDA scale-up will result in decreased marginal costs (the cost of one additional treatment) by assuming there to be spare capacity among fixed costs (cars, equipment, etc.). However, in some areas, additional capital may be required and, therefore, marginal costs would increase. In such a case, accelerating eradication might not be the most cost-saving option in the short term.

Additionally, our analysis does not take into account the cost for certifying elimination on a country level nor the activities involved in globally assessing whether eradication has been achieved. As currently experienced by the Global Polio Eradication Initiative, the costs for finding and ascertaining the last cases to reach eradication are substantial [40, 41]. While our analysis does not consider the costs associated with finding the last cases of LF infection, the unit costs for the final populations treated in the eradication I scenario are orders of magnitude higher than the average unit costs—beyond 1,700 USD per person treated. This is due to fewer people to be treated (the denominator of the calculation) rather than higher overall costs of running the program. It has previously been recognized that when the cost of disease eradication comes within reach, the unit costs associated with prevention become decreasingly attractive. However, at that point, it is crucial not to lose momentum, nor investment, otherwise there is great risk of failure [42, 43]. Developing realistic cost projections from the start of the program could help mitigate the risk of donor fatigue towards the endgame of disease eradication. Further, when disease eradication is within reach, shifting the focus from unit costs per person treated to the costs per case averted may also help to sustain global commitments [44].

Our analysis found a 15% increase in financial costs to treat an area of hyper L. loa endemicity in the DRC. This estimate does not take into account increases in advocacy and communication in these areas, which may be necessary to achieve the targeted levels of coverage. Further, the costs for medical transport and additional medications that might be needed to treat patients suffering from severe adverse events have not been incorporated in this analysis. Costing studies for such post-MDA response activities have not previously been carried out, though such costs are likely to vary by the incidence of SAEs, the geographic location, and the intensity of response required. If substantial and significant response is required in many areas, implementing the eradication scenarios would certainly result in higher costs than projected in this baseline study.

A number of methodological uncertainties in our study must be mentioned. Country-specific cost data was mostly unavailable and, consequently, was largely imputed from Ugandan data. By extrapolating cost data across countries and regions, we inherently made the assumption that each LF endemic country implements the GPELF strategy as in Uganda (for example, using volunteer CDDs, similar amount of trainings, etc.). Moreover, we assumed that the number of resources required to carry out the PELF for a certain population in Uganda remains relatively constant (varying by +/-10%) both across time and across countries. As described above, the variation assumed in our model was included probabilistically in both unit costs and quantities.

We chose to have modest uncertainty assumptions, assuming a variation of 10% probabilistically for both costs and quantity estimates. Though 10% variation has a large impact on the individual unit costs, the impact on total costs was less than expected. As such, we chose to increase the variability to 30% during additional uncertainty analyses. However, this resulted in only a modest increase in the uncertainty intervals (see S3 Table and S1 Fig). It is, therefore, likely that the narrow uncertainty intervals found in this analysis are the result of assumptions made during earlier analyses to estimate the number of annual rounds of treatment required to interrupt LF transmission. Namely, we assumed the required number of annual rounds of treatments to be fixed at the upper bound of our model-based projections in order to provide as conservative an estimate as we could. Varying the estimated number of treatments across their credible interval may have instead provided a more complete picture.

Additionally, our MDA round estimates are considerably longer than the 4–6 years currently specified by the GPELF, instead ranging from 6 rounds of MDA in areas where Anopheles spp are the primary vector, treatment is through DEC+ALB, and the baseline MF prevalence is 5–10%, to 15 rounds in areas where Culex spp are the primary vector, IVM+ALB is the treatment provided and the baseline mf prevalence is 15–20% [19, 45]. These extended MDA durations have a number of implications, but most relevant in terms of costing is that both the financial and economic estimates are likely to be much more conservative than estimates developed using an assumed 4–6 years of MDA, since the overall costs are heavily dependent on the duration of the program. Our approach, therefore, resulted in conservative estimates at the cost of capturing more realistic uncertainty intervals. In our baseline analysis we chose to make rather crude assumptions of independence between inputs as well as assumptions of the uncertainty and distributions of the model inputs. For the purposes of assessing the relative costs of scaling up MDA at different rates we felt that this was sufficient in order to avoid introducing increased complexity, and consequently, further uncertainty in our model.

Ideally, LF-specific expenditure data would have been collected in all 72 LF-endemic countries. Having such data would have also allowed for an important assessment on the variance of costs from the beginning stages of implementation towards the ‘end game’ of reaching surveillance. However, 14 of these endemic countries have never carried out MDA for LF [13]. Moreover, undertaking a study that accurately collected such data would potentially begin to rival the time and cost of running the PELF programs in many of the countries to begin with. Further, a recent study by Brady et al. found no significant difference between grant budgets and actual expenditures when analyzing costs of Transmission Assessment Surveys [46]. Thus, despite the large number of assumptions inherent in our approach, our costing model allowed for the development of comparable cost estimates on a country, regional, and global level.

We conducted a probabilistic sensitivity analysis in order to overcome some of the limitations inherent in our costing parameters. In order to provide more robust estimates on the costs of achieving elimination and eradication, costing studies in areas with the highest burden of LF could be undertaken in order to reduce the level of uncertainty in costs. While improved cost data could be used to inform policy and improve planning, the cost of acquiring additional data should be weighed against the value of such data [47]. An additional use for the results found in our economic analyses, therefore, could be to assess the value of additional investments for LF, including the collection of expenditure data, as well as investments in diagnostics, drugs, and surveillance tools to help advance LF eradication.

Our findings on the costs to eliminate and eradicate LF represent very achievable investments. Our cost estimates would have likely been even lower, though, if we assumed some level of integration or cost-sharing between other disease initiatives. Many countries have, in fact, integrated similar activities across vertical programs, including onchocerciasis, trachoma, and schistosomiasis, and others have paired drug delivery for MDA with other community distribution campaigns, including insecticide treated nets (ITNs) for malaria and ongoing vaccine programs. In so doing, the overall costs per program, indeed, generally decreased and efficiency reportedly improved [2, 48–51]. However, it is important to note that, historically, other disease elimination programs that have proven to be successful have generally proceed as vertical programs [52]. Additionally, given the cost involved with each round of MDA, it could be cost saving to undertake TAS sooner in order to assess whether the interruption of transmission had been achieved. However, this approach could prove to be a difficult balance, since prematurely stopping MDA could result in resurgence of infection [53], ultimately leading to an increase in the cost of reaching eradication.

We further did not include the cost of vector control in this costing study, assuming instead the cost to be covered by other disease control programs (for example: malaria or dengue control). While vector control is not a core strategy of the GPELF in non *l*. *loa* endemic areas, previous studies have indicated that including vector control could decrease the number of MDA rounds needed to interrupt transmission, thereby reducing the overall duration of the program [54], and potentially also the cost.

A comparison of our costs against other costs is important for validation, though challenging due to differing methodologies. Though not inclusive of overhead costs, a study from two states in Nigeria found the cost associated with conducting MDA for the prevention of LF to be between 0.02 USD and 0.12 USD per treatment delivered [55]. A multi-country costing study conducted by Goldman et. al found unit financial costs to range from 0.06 USD in Burkina Faso to 2.23 USD in Haiti [34]. A separate study in Haiti reported the cost per person treated to be 1.44 USD [56]. Thus, in comparison to other MDA costing studies, our average unit financial cost estimates are well within the range of previously reported studies.

Knowing the global costs of the program will help decision makers assess the feasibility and rationale of investing in LF eradication, while simultaneously helping to facilitate planning and the development of strategies and policies. However, successfully eradicating LF depends on more than the monetary investment. Political will, continued community ownership, and the feasibility of the campaign all need to be taken into account [57]. However, if successful, disease eradication not only results in innumerable long-term health benefits, but also savings to the health system, gains in productivity, and improvements in social justice [17, 58]. The decision of whether disease eradication should be pursued, therefore, needs to be approached with a comprehensive understanding of the many complex issues at play.

## Supporting information

S1 File Description of sensitivity analyses. (DOCX)

S1 Fig Total median financial costs, 30% uncertainty. (DOC)

S2 Fig Two-way sensitivity analysis: Capacity strengthening vs. salary. (DOC)

S3 Fig Two-way sensitivity analysis: Advocacy vs. distance. (DOC)

S1 Table Key features of the proposed scenarios for the elimination and eradication of LF. (DOC)

S2 Table Parameters used in the probabilistic sensitivity analysis. (DOC)

S3 Table Total median financial costs by scenario and associated 95% credible intervals. (DOC)

S4 Table Two-way sensitivity analysis: Capacity strengthening vs. salary. (DOC)

S5 Table Two-way sensitivity analysis: Advocacy vs. distance. (DOC)
