# Supplementary material for: How much will it cost to eradicate lymphatic filariasis? An analysis of the financial and economic costs of intensified efforts against lymphatic filariasis
Source: PLoS Negl Trop Dis. 2017 Sep 26;11(9):e0005934. doi: 10.1371/journal.pntd.0005934 (PMC5630187; doi:10.1371/journal.pntd.0005934)
Supplement: S1 File — (DOCX) [file pntd.0005934.s001.docx]

**Description of Sensitivity Analyses**

###

### **Probabilistic Sensitivity Analysis**

Our micro-costing model was built up using resource quantities and associated unit costs. Following guidance from Briggs et. al, we undertook a probabilistic sensitivity analysis (PS) assuming gamma distributions, parameterized as gamma(α,β), for all unit cost inputs.. The deterministic value was assumed to be the sample mean (µ), with variance *s.* Which follows:

$\mu=$ αβ, $s^{2}=\alpha\beta^{2}$

$\alpha=\frac{\mu^{2}}{s^{2}}$*,* $\beta=\frac{s^{2}}{\mu}$

[151]

We assumed 10% uncertainty across all parameters. Supplement Table 2 lists all parameters considered along with their deterministic value, calculated standard error, alpha, and beta estimates.

**Additional Sensitivity Analyses**

**Variability.** Our initial analysis included 10% variability on all input parameters. In order to explore the impact of incorporating broader uncertainty in our analysis, we reran the costing model for 500 iterations with the assumption of 30% uncertainty in all input parameters. As anticipated, this change has resulted in wider uncertainty intervals (see S3 Table, S1 Fig).

**Correlation.** To assess the robustness of our overall results, we have also carried out two, two-way sensitivity analyses to examine possible correlation among input parameters. Each two-way sensitivity analysis examined two variables at a time and considered, simultaneously, the impact of their changes on the overall results.

We chose to first explore the possible interdependence of capacity strengthening, the activity that made up more than 50% of the overall costs in the baseline analysis, and salaries assuming -15%, +15% and +30% change in the baseline costs of all salaries and capacity strengthening activities. Varying salaries and capacity strengthening simultaneously resulted in cost ranges in the elimination (comparator) scenario between $775m-$834m USD when assuming a 15% decrease in both the costs of capacity strengthening and salaries, $1,074m-1,167m USD when assuming a 30% increase in costs of capacity strengthening and salaries. As shown in the figure below, costs fall into the highest range (orange) when capacity strengthening activities are assumed to increase by 30%, or when capacity strengthening increases by +15% and salaries increase by at least 15% (see S4 Table, S2 Fig)

In the second two-way sensitivity analysis, we examined the robustness of our overall results when simultaneously varying the costs of advocacy with distance, as we expected these variables to have the most likely occurrence of covariance, again assuming a change of -15%, +15% and +30% in the baseline costs of the variables under study. In assuming a decrease in baseline costs of 15% among advocacy activities and distances travelled, the overall cost of the elimination scenario ranged from $836m-$908m. Assuming a 30% increase in these parameters resulted in an overall cost to the elimination scenario of $926m-$1,006m. In looking at the visual representation of these results below, it becomes clear that, within the changes considered, costs are more likely to fall with the 900m-950m range. Further, when the cost of advocacy activities decrease by -15%, the overall costs fall into the lowest range (red: $850m-900m), even when distances increase by 30%. Similarly, when advocacy costs are assume to increase by 30%, overall financial costs fall into the highest range (purple: $950m-1,000m), even when distances decrease by -15%. Importantly, movement from one category of total costs to another (higher or lower) occurred at any change of distance (see S5 Table, S3 Fig).
