# Supplementary figures and images for: How much will it cost to eradicate lymphatic filariasis? An analysis of the financial and economic costs of intensified efforts against lymphatic filariasis
Source: PLoS Negl Trop Dis. 2017 Sep 26;11(9):e0005934. doi: 10.1371/journal.pntd.0005934 (PMC5630187; doi:10.1371/journal.pntd.0005934)

**S1 Figure: Total median financial costs, 30% uncertainty**


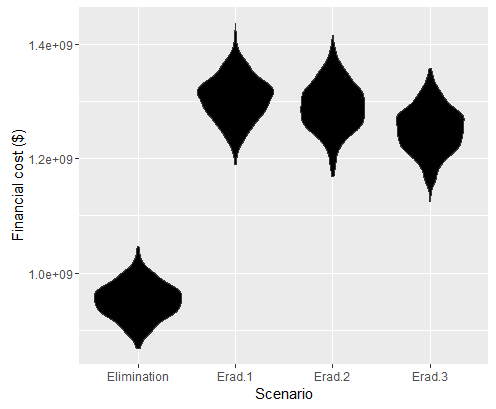

Supplement: S1 Fig — (DOC) [file pntd.0005934.s002.doc]

**S2 Figure: Two-way sensitivity analysis: Capacity strengthening vs. salary**

**
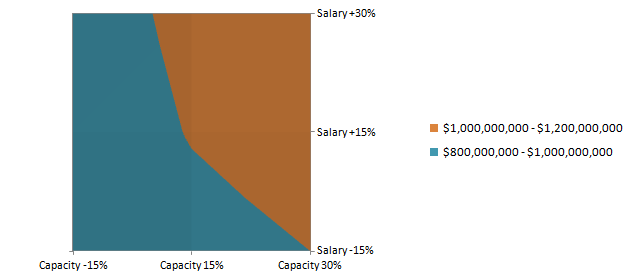
**

Supplement: S2 Fig — (DOC) [file pntd.0005934.s003.doc]

**S3 Figure: Two-way sensitivity analysis: Advocacy vs. distance**


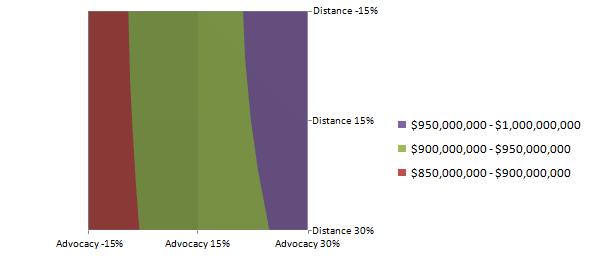

Supplement: S3 Fig — (DOC) [file pntd.0005934.s004.doc]
