# Supplementary material for: How much will it cost to eradicate lymphatic filariasis? An analysis of the financial and economic costs of intensified efforts against lymphatic filariasis
Source: PLoS Negl Trop Dis. 2017 Sep 26;11(9):e0005934. doi: 10.1371/journal.pntd.0005934 (PMC5630187; doi:10.1371/journal.pntd.0005934)
Supplement: S1 Table — (DOC) [file pntd.0005934.s005.doc]

S1 Table: Key features of the proposed scenarios for the elimination and eradication of LF

|  | **Elimination (comparator)** | **Eradication I** | **Eradication II** | **Eradication III** |
| --- | --- | --- | --- | --- |
| **Intervention** | Annual MDA* | Annual MDA* | Annual MDA* | Annual MDA* |
| **Coverage rate** | 85% | 85% | 85% | 85% |
| **Start year** | 2014 | 2014 | 2014 | 2014 |
| **Countries considered** | All LF endemic countries that have previously conducted MDA^¥^ | All LF endemic countries^¥^, including all countries co-endemic for *L. loa* | All LF endemic countries^¥^, including all countries co-endemic for *L. loa* | All LF endemic countries^¥^, including all countries co-endemic for *L. loa* |
| **Rate of scale-up** | Countries with previous MDA continue at same rate as historically | Countries with previous MDA continue at same rate as historically, countries without previous progress begin at ‘average’ rate (10% of at-risk population added to MDA schedule annually) | All countries add 20% of their at-risk populations to MDA schedule annually | All countries treat 100% of their at-risk populations annually |
| **Estimated final year of MDA** | 2050 | 2050 | 2032 | 2028 |
| **Number of treatments necessary (millions)** | 3,409  (3,185-3,538) | 4,667 (4,419-4,904) | 4,369 (4,133-4,594) | 4,159 (3,924-4,382) |

^*^Except in areas where L. loa prevalence exceeds 40%.

^¥^Assuming country requires MDA
