# Supplementary material for: How much will it cost to eradicate lymphatic filariasis? An analysis of the financial and economic costs of intensified efforts against lymphatic filariasis
Source: PLoS Negl Trop Dis. 2017 Sep 26;11(9):e0005934. doi: 10.1371/journal.pntd.0005934 (PMC5630187; doi:10.1371/journal.pntd.0005934)
Supplement: S2 Table — (DOC) [file pntd.0005934.s006.doc]

S2 Table: Parameters used in the Probabilistic Sensitivity Analysis

| Parameters | Deterministic Value | Standard Error | Alpha | Beta |
| --- | --- | --- | --- | --- |
| **Per diem rates** |  |  |  |  |
| Case management specialist | $40.00 | 4.000 | 100 | 0.4000 |
| Community moderator | $4.68 | 0.468 | 100 | 0.0468 |
| CDD - training | $1.17 | 0.117 | 100 | 0.0117 |
| CDD - providing data | $0.78 | 0.078 | 100 | 0.0078 |
| Clinical officer | $19.50 | 1.950 | 100 | 0.1950 |
| Community leaders | $4.68 | 0.468 | 100 | 0.0468 |
| Data entry clerk | $78.00 | 7.800 | 100 | 0.7800 |
| District representative | $20.00 | 2.000 | 100 | 0.2000 |
| District vector control officer | $19.50 | 1.950 | 100 | 0.1950 |
| District support staff: district guide | $4.68 | 0.468 | 100 | 0.0468 |
| District support staff: local councillor (teachers) | $1.95 | 0.195 | 100 | 0.0195 |
| District support staff: local councillor (mobilizer) | $4.29 | 0.429 | 100 | 0.0429 |
| Driver | $21.45 | 2.145 | 100 | 0.2145 |
| Health promotion and education officer | $41.26 | 4.000 | 100 | 0.4000 |
| IT consultant | $38.87 | 4.000 | 100 | 0.4000 |
| M&E officer | $38.14 | 4.000 | 100 | 0.4000 |
| Minister of health | $43.33 | 5.000 | 100 | 0.5000 |
| National trainers | $38.50 | 4.000 | 100 | 0.4000 |
| NTD Coordinator | $48.79 | 4.000 | 100 | 0.4000 |
| Parish supervisors | $ 4.74 | 0.468 | 100 | 0.0468 |
| Partners | - |  |  |  |
| Procurement officer | $41.36 | 4.000 | 100 | 0.4000 |
| Programme managers | $33.25 | 4.000 | 100 | 0.4000 |
| Religious leaders | $4.68 | 0.468 | 100 | 0.0468 |
| Senior scientist | $42.90 | 4.290 | 100 | 0.4290 |
| Social scientists | $40.00 | 4.000 | 100 | 0.4000 |
| Sub county supervisors | $4.68 | 0.468 | 100 | 0.0468 |
| Support staff, rural | $4.68 | 0.468 | 100 | 0.0468 |
| Support staff, urban | $10.00 | 1.000 | 100 | 0.1000 |
| Technician | $39.00 | 3.900 | 100 | 0.3900 |
| ToTs from District Health Office | $4.68 | 0.468 | 100 | 0.0468 |
| ToTs from sub county | $19.50 | 1.950 | 100 | 0.1950 |
| Village chief | $4.68 | 0.468 | 100 | 0.0468 |
| **Materials and supplies** |  |  |  |  |
| Accommodation - rural | $11.70 | 1.170 | 100 | 0.1170 |
| Accommodation - urban | $100.00 | 10.000 | 100 | 1.0000 |
| Advocacy booklets | $1.50 | 0.150 | 100 | 0.0150 |
| Air time | $19.50 | 1.950 | 100 | 0.1950 |
| Alcohol swab (packet of 100) | $4.68 | 0.468 | 100 | 0.0468 |
| Bags for CDDS | $0.39 | 0.039 | 100 | 0.0039 |
| Banners | $25.00 | 2.500 | 100 | 0.2500 |
| Batteries (size D) | $1.17 | 0.117 | 100 | 0.0117 |
| Billboards | $200.00 | 20.000 | 100 | 2.0000 |
| Blood lancets (box of 200 pieces) | $4.68 | 0.468 | 100 | 0.0468 |
| Box files | $2.54 | 0.254 | 100 | 0.0254 |
| Broadcast spots on radio | $234.00 | 23.400 | 100 | 2.3400 |
| Broadcast spots on TV (1 hour) | $390.00 | 39.000 | 100 | 3.9000 |
| Capillary tubes (60 ul), 100 pieces | $9.75 | 0.975 | 100 | 0.0975 |
| Coartem or (ARCO) | $1.76 | 0.176 | 100 | 0.0176 |
| Co-trimazole Tablets (1000) | $8.27 | 0.827 | 100 | 0.0827 |
| Cotton wool (500 gram roll) | $1.95 | 0.195 | 100 | 0.0195 |
| Counter book (4 quire) | $2.34 | 0.234 | 100 | 0.0234 |
| Dettol soap (100 grams) | $0.98 | 0.098 | 100 | 0.0098 |
| Gauze | $3.14 | 0.314 | 100 | 0.0314 |
| Gimesa stain | $90.65 | 9.065 | 100 | 0.9065 |
| Glass slides (50 pcs) | $2.54 | 0.254 | 100 | 0.0254 |
| Gloves (Disposable Rubber) | $4.88 | 0.488 | 100 | 0.0488 |
| Hall rental, capital | $150.00 | 15.000 | 100 | 1.5000 |
| Hall rental, other | $19.50 | 1.950 | 100 | 0.1950 |
| Heparine coated container(200µl) | $0.20 | 0.020 | 100 | 0.0020 |
| Ibuprofen Tablets (1000) | $3.90 | 0.390 | 100 | 0.0390 |
| Internet and accessories | $1,000.00 | 100.000 | 100 | 10.0000 |
| Labels (Packet of 200 labels) | $1.95 | 0.195 | 100 | 0.0195 |
| Laundry soap - bar | $1.17 | 0.117 | 100 | 0.0117 |
| Laundry soap -Detergent powder (1 kg) | $3.12 | 0.312 | 100 | 0.0312 |
| Magnesium trisilicate (1000) | $2.93 | 0.293 | 100 | 0.0293 |
| Match box (10 pieces) | $0.39 | 0.039 | 100 | 0.0039 |
| Meals, capital | $7.80 | 0.780 | 100 | 0.0780 |
| Megaphone | $45.00 | 4.500 | 100 | 0.4500 |
| Mobile phones | $80.00 | 8.000 | 100 | 0.8000 |
| Neomycin ointment (12) | $7.02 | 0.702 | 100 | 0.0702 |
| Paracetamol Tablets (1000) | $3.90 | 0.390 | 100 | 0.0390 |
| Paraffin | $0.94 | 0.094 | 100 | 0.0094 |
| Pens (packets of 50) | $0.20 | 0.020 | 100 | 0.0020 |
| Pencils (HB) dozens | $0.02 | 0.002 | 100 | 0.0002 |
| Permanent marker | $2.60 | 0.260 | 100 | 0.0260 |
| Photocopying paper | $6.63 | 0.663 | 100 | 0.0663 |
| Pipette tips | $0.39 | 0.039 | 100 | 0.0039 |
| Pamphlets for districts | $0.39 | 0.039 | 100 | 0.0039 |
| Posters | $1.17 | 0.117 | 100 | 0.0117 |
| Refreshments, capital | $5.00 | 0.500 | 100 | 0.0500 |
| Refreshments, rural | $1.37 | 0.137 | 100 | 0.0137 |
| Reproduction of advocacy materials | $0.10 | 0.010 | 100 | 0.0010 |
| Safety boxes (Sharps container) | $0.39 | 0.039 | 100 | 0.0039 |
| Sentinel site forms | $0.03 | 0.003 | 100 | 0.0003 |
| Stationary | $1.17 | 0.117 | 100 | 0.0117 |
| Survey forms | $0.03 | 0.003 | 100 | 0.0003 |
| Sweets | $5.85 | 0.585 | 100 | 0.0585 |
| Test kits - Binax Now(25 test kits) | $5.48 | 0.548 | 100 | 0.0548 |
| Trash bags (50 pieces) | $1.95 | 0.195 | 100 | 0.0195 |
| Trash containers (50 pieces) | $1.95 | 0.195 | 100 | 0.0195 |
| Toilet paper (20 roll carton) | $4.29 | 0.429 | 100 | 0.0429 |
| T-shirts | $3.90 | 0.390 | 100 | 0.0390 |
| **Activities** |  |  |  |  |
| Data cleaning/ Entry Clerk/Analysis | $975.00 | 97.500 | 100 | 9.7500 |
| Institution review clearance fees | $210.60 | 21.060 | 100 | 2.1060 |
| Report writing (lump sum) | $195.00 | 19.500 | 100 | 1.9500 |
| Slide reading | $0.39 | 0.039 | 100 | 0.0039 |
| Data analysis | $195.00 | 19.500 | 100 | 1.9500 |
| **Demographics** |  |  |  |  |
| # of people per district | 279,089 | 27909 | 100 | 2,790.8900 |
| # people per subdistrict | 18,006 | 1801 | 100 | 180.0600 |
| # parishes per district | 64 | 6 | 100 | 0.6400 |
| # people per village | 730 | 73 | 100 | 7.3000 |
| # schools per district | 209 | 21 | 100 | 2.0894 |
