# Supplementary material for: How much will it cost to eradicate lymphatic filariasis? An analysis of the financial and economic costs of intensified efforts against lymphatic filariasis
Source: PLoS Negl Trop Dis. 2017 Sep 26;11(9):e0005934. doi: 10.1371/journal.pntd.0005934 (PMC5630187; doi:10.1371/journal.pntd.0005934)
Supplement: S3 Table — (DOC) [file pntd.0005934.s007.doc]

**S3 Table: Total median financial costs by scenario and associated** 95% credible intervals

|  | Elimination Scenario | Eradication I Scenario | Eradication II Scenario | Eradication III Scenario |
| --- | --- | --- | --- | --- |
| 10% uncertainty | $929.2m  (890.5m-965.4m) | $1,289m  ($1,239m-$1,337m) | $1,273m  ($1,223m-$1,322m) | $1,235m  ($1,183m-$1,284m) |
| 30% uncertainty | $951.3m  ($896.9m-$1,002.7m) | $1,307m  ($1,238m-$1,368m) | $1,291m  ($1,223m-$1,361m) | $1,251m  ($1,180m-$1,316m) |
