# Supplementary material for: How much will it cost to eradicate lymphatic filariasis? An analysis of the financial and economic costs of intensified efforts against lymphatic filariasis
Source: PLoS Negl Trop Dis. 2017 Sep 26;11(9):e0005934. doi: 10.1371/journal.pntd.0005934 (PMC5630187; doi:10.1371/journal.pntd.0005934)
Supplement: S4 Table — (DOC) [file pntd.0005934.s008.doc]

**S4 Table: Two-way sensitivity analysis: Capacity strengthening vs. s**alary

|  | **Capacity Strengthening -15%** | **Capacity Strengthening +15%** | **Capacity Strengthening +30%** |
| --- | --- | --- | --- |
| **Salary -15%** | $804m ($775m-$834m) | $935m ($89m-$973m) | $1,001m ($959m-$1,040m) |
| **Salary +15%** | $869m ($835m-$905m) | $1,011m ($967m-$1,051m) | $1,080m ($1,034m-1,123m) |
| **Salary +30%** | $902m ($864m-$935m) | $1,048m ($1,004m-$1,090m) | $1,120m ($1,074m-$1,167m) |
