# Supplementary material for: How much will it cost to eradicate lymphatic filariasis? An analysis of the financial and economic costs of intensified efforts against lymphatic filariasis
Source: PLoS Negl Trop Dis. 2017 Sep 26;11(9):e0005934. doi: 10.1371/journal.pntd.0005934 (PMC5630187; doi:10.1371/journal.pntd.0005934)
Supplement: S5 Table — (DOC) [file pntd.0005934.s009.doc]

**S5 Table: Two-way sensitivity analysis: Advocacy vs. distance**

|  | **Advocacy -15%** | **Advocacy +15%** | **Advocacy +30%** |
| --- | --- | --- | --- |
| **Distance -15%** | $873m ($836m-$908m) | $931m ($891m-$969m) | $960m ($923m-$1,000m) |
| **Distance +15%** | $877m ($840m-$910m) | $935m ($896m-$976m) | $966m ($923m-$1,005m) |
| **Distance +30%** | $878m ($844m-$914m) | $938m ($901m-$976m) | $967m ($926m-$1,006m) |
